# Hospitals from a Statistical Point of View

**Published:** 1890-12-20

**Authors:** 


### PASSING TOPICS.

### HOSPITALS FROM THE STATISTICAL POINT OF VIEW.

The address lately delivered by the President of the Royal Statistical Society deals largely with the hospital question, and some of Dr. Mouatt's observations under this head are worthy of a wider circulation than perhaps they are like to attain. The general reader finds but little to tempt him in figures; to him they are caviare, and when it has been observed with a show of fine disdain that "anything may be proved by statistics," the average controversialist is well satisfied that he has the best of the argument. It is true that figures lend themselves a little too readily to feats of jugglery, and may be made at times to support propositions more than doubtful, but this is the fault not of the material but of the manipulation. Figures left to themselves are doughty opponents, and, if possible, it is best to have them on our own side.

The statistics of our hospitals are capable of plain treatment, and might be compiled in a way easily to be understood. The mystification which ensues immediately they are examined is due primarily to the want of uniform methods among institutions, and after that, to the overweening desire on the part of certain petty organizations to impress the beholder with a sense of vastness unwarranted by the real proportions of the work. We all know the effect of viewing approaching objects through the medium of a fog—how they become invested with vague and shadowy outlines suggestive of size undreamed of in actual life, and it is some such phantasmagoria as this well spread with colour and addressed powerfully to the imagination of the uninformed which is continually danced before the eyes of the donation-giving public by the promoters of insignificant and questionable "charities."

The saddest feature is that the actualities of hospital deficiency are solid enough to fill their parts independently of artificial aids. They are unmistakably in evidence, but the public eye refuses to see them. On the one hand, there is much puffing of the fur and feathers of pigmy organisms with scarcely an ounce of flesh to recommend them; on the other, we have the spectacle of certain organised efforts to hide the realities of suffering, because, forsooth, the undeserving obtain a share of what is provided for its relief.

The President of the Statistical Society is firmly persuaded of "the need of a radical change of procedure and practice in the provision of immediate and effective hospital aid," and regards it as "one of the most important economic and social matters that press for early solution and legislative action." We have no shadow of doubt that this is the view which must be taken by any competent person who investigates the facts for himself. "I think," adds Dr. Mouatt, "it may not be unreasonably assumed, from all that has been said and written on the subject for many years past, that the present hospital system of London is gradually breaking down from sheer physical inability to deal with the unprecedented growth, and consequent changed conditions of life in the metropolis." These words involve a grave arraignment of our hospital system, not of the hospitals, which would be a very different thing, and if they express the deliberate opinion of one who has studied the question carefully, and is in a position to give judgment, may we not join with the speaker in a hope, as we wish we could share the belief, that there is some likelihood now of the legislative action so imperatively called for? AGRICOLA.

